# Acetyltransferases GCN5 and PCAF Are Required for B Lymphocyte Maturation in Mice

**DOI:** 10.3390/biom12010061

**Published:** 2021-12-31

**Authors:** Valentyn Oksenych, Dan Su, Jeremy A. Daniel

**Affiliations:** 1 The NNF Center for Protein Research, Faculty of Health and Medical Sciences, University of Copenhagen, Blegdamsvej 3B, 2200 Copenhagen, Denmark; dan.su@jiangnan.edu.cn; 2 Laboratory Center, Department for Cancer Research and Molecular Medicine (IKOM), Norwegian University of Science and Technology, Erling Skjalgssons Gate 1, 7491 Trondheim, Norway

**Keywords:** KAT2A, KAT2B, mice, acetyltransferase, B cell, lymphocyte, class switching

## Abstract

B lymphocyte development has two DNA recombination processes: V(D)J recombination of the immunoglobulin (*Igh*) gene variable region, and class switching of the *Igh* constant regions from IgM to IgG, IgA, or IgE. V(D)J recombination is required for the successful maturation of B cells from pro-B to pre-B to immature-B and then to mature B cells in the bone marrow. CSR occurs outside of the bone marrow when mature B cells migrate to peripheral lymphoid organs, such as spleen and lymph nodes. Both V(D)J recombination and CSR depend on an open chromatin state that makes DNA accessible to specific enzymes, recombination activating gene (RAG), and activation-induced cytidine deaminase (AID). Acetyltransferases GCN5 and PCAF possess redundant functions acetylating histone H3 lysine 9 (H3K9). Here, we generated a mouse model that lacked both GCN5 and PCAF in B cells. Double-deficient mice possessed low levels of mature B cells in the bone marrow and peripheral organs, an accumulation of pro-B cells in bone marrow, and reduced CSR levels. We concluded that both GCN5 and PCAF are required for B-cell development in vivo.

## 1. Introduction

The development of B lymphocytes starts in the bone marrow where progenitor (pro)-B cells using recombination-activating genes (RAG) generate DNA double-strand breaks (DSBs) and initiate V(D)J recombination [1]. In maturating B cells, the V(D)J recombination process is genetic recombination of *variable (V)*, *diversity (D)*, and *joining (J)* gene segments arranging into a newly formed VDJ part of immunoglobulin gene (*Ig*) [2,3,4,5,6]. Following V(D)J recombination, B cells develop from pro-B cells expressing specific markers cluster of differentiation 19 (CD19), B220/CD45, and CD43 (CD19+B220+CD43+) to pre-B cells (CD19+B220+CD43−), immature B (CD19+B220+IgM+, low immunoglobulin M, IgM) and mature B (CD19+B220+IgM+, high IgM) cells in bone marrow [2]. Mature B lymphocytes leave the bone marrow and migrate to periphery-populating spleen and lymph nodes through the blood.

Then, mature B cells initiate another DNA recombination process to change the constant regions of immunoglobulin genes, referred to as class switch recombination (CSR). During the CSR in mice, IgM is replaced by IgG3, IgG1, IgG2a, IgG2b, IgE, or IgA [2]. The CSR is initiated by nonproductive transcription known as a germline transcription (GLT), which is needed to separate two DNA strands. Single-stranded DNA is then targeted by activation-induced cytidine deaminase (AID), a B lymphocyte-specific enzyme deaminating cytosine to uracil (C to U). Then, uracil DNA N-glycosylase (UNG) removes uracil from DNA, leading to single-strand break formation (SSB) [7]. Two SSBs facing each other then form a DSB and allow for recombination [2,3,4,5,8]. Both V(D)J and CSR can be regarded as processes following fundamentally similar strategies of genomic recombination [9,10].

DSBs formed during the V(D)J recombination and CSR are recognized, processed, and repaired by the nonhomologous end-joining pathway (NHEJ), and initiate a more complex signaling and chromatin modification pathway known as DNA damage response (DDR) [2,3,4,5]. The NHEJ is initiated when the Ku70 and Ku80 heterodimer (Ku) recognizes and binds the DSBs. Ku serves as a platform for downstream factors including DNA-dependent protein kinase catalytic subunit (DNA-PKcs), *X*-ray repair cross-complementing protein 4 (XRCC4), XRCC4-like factor (XLF), the paralogue of XRCC4 and XLF (PAXX), a modulator of retroviral infection (MRI), and DNA ligase 4 (LIG4) [3,4,5,11]. There are additional factors that are sometimes optional for NHEJ, including Artemis with nuclease activity required for processing hairpin-sealed DNA ends and overhangs [2,5,12].

One type of DDR pathway acts downstream of the ataxia telangiectasia mutated (ATM) protein kinase, which is activated by DSBs and then phosphorylates multiple substrates, including NHEJ and DDR factors. ATM phosphorylates histone H2AX, which in turn recruits the mediator of DNA damage checkpoint 1 (MDC1) and facilitates the accumulation of really interesting new gene (RING) finger motif (RNF) 8 and RNF168 ubiquitin ligases, and then the p53-binding protein (53BP1). The phosphorylation of H2AX is related to the acetylation of histones, including histone H3K9. In particular, histone acetylation relies on ATM-dependent H2AX phosphorylation and SWI/SNF chromatin remodeling factors [13]. The acetylation of histone H3K9 is mediated by GCN5 and PCAF [13,14].

There is a complex genetic interaction associated with the functional redundancy between NHEJ factors [3,4], including the following pairs: DNA-PKcs/XLF [5,15,16,17], PAXX/XLF [18,19,20,21,22,23,24], and MRI/XLF [25,26]. Moreover, there is a genetic interaction between NHEJ and DDR pathway factors ATM/XLF and H2AX/XLF [27], MDC1/XLF [28], RNF8/XLF and RNF168/XLF [29], 53BP1/XLF [30,31], and others [3,4]. Moreover, acetyltransferases GCN5 and PCAF are redundant in promoting histone H3 lysine K9 acetylation [32].

General control nondepressible 5 (GCN5) acetyltransferase is also known as lysine acetyltransferase (KAT) 2A. The germline inactivation of GCN5 in mice resulted in early embryonic lethality due to the role of the protein in neurogenesis [32]. GCN5 is functionally redundant with another acetyltransferase, KAT2B, also referred to as p300/CBP-associated factor (PCAF). While the inactivation of the *Pcaf* gene in mice has no detectable phenotype, the double knockout of *Gcn5/Pcaf* genes resulted in even earlier embryonic lethality than that in *Gcn5^−/−^* mice [32]. Because histone H3K9 acetylation works downstream of ATM and H2AX in DDR [13], one could propose that GCN5, PCAF, or both enzymes are required for lymphocyte development in vivo. However, the embryonic lethality of *Gcn5^−/−^* and *Gcn5^−/−^Pcaf^−/−^* mice [32] challenged studies. To overcome the obstacle, we developed a complex mouse model when *Pcaf* gene was germline-inactivated [32] while floxed *Gcn5* gene [33] was conditionally inactivated in B-cell lineages by CRE recombinase expressed under *Cd19* promoter [34]. To sort out CRE-positive and -negative cells, we used *Rosa26-stop-YFP* knockin, which only expressed YFP following CRE activation [35].

Here, we found that GCN5 and PCAF acetyltransferases are functionally redundant during early B-cell maturation, while GCN5 is required for robust CSR.

## 2. Materials and Methods

### 2.1. Mice

*Gcn5^f/f^* [33], *Pcaf^+/−^* [32], *Cd19^Cre+^* [34] (no. 006785; The Jackson Laboratory, Bar Harbor, ME, USA), *Rosa26-stop-YFP^+^* [35] (no. 006148, The Jackson Laboratory, Bar Harbor, ME, USA), and *Aid^−/−^* [36] mice were previously described. Mice used for experiments were between 8 and 12 weeks of age. All experiments were performed in compliance with the Danish Working Environment Authority, the Danish Animal Experiment Inspectorate, the Department of Experimental Medicine (University of Copenhagen, Copenhagen, Denmark), and the Animal Resources Care Facility of Norwegian University of Science and Technology (NTNU, Trondheim, Norway).

### 2.2. Flow Cytometry

Flow cytometry experiments were performed as we described earlier [25,28,37,38,39]. In particular, we used fluorescent antibodies recognizing the proteins described below. B220 (PE-CF594, FITC, Alexa Fluor 700; all clone RA3-6B2, BD Bioscience, Franklin Lakes, NJ, USA). IgM (PerCP-eFluor 710, APC-eFluor 780, APC, FITC; all clone II/41, eBioscience, Santa Clara, CA, USA). IgG1 (PE, MOPC-21, Biolegend, SanDiego, CA, USA). IgG3 (PE, MG3-35, Biolegend, San Diego, CA, USA). CD3-APC (Biolegends, USA, #100312). CD19 (Alexa Fluor 700, APC eFluor 780, both clone 1D3, eBioscience, Santa Clara, CA, USA). CD43 (APC and PE-Cy7, both clones S7, BD Bioscience, Franklin Lakes, NJ, USA).

### 2.3. Class Switch Recombination

The CSR was performed as we described earlier [15,27,38,39,40,41,42].

### 2.4. Western Blot

The Western blot procedures were performed as we described earlier [28,37,38,43]. Briefly, cells were lysed for 30 min on ice in a radioimmunoprecipitation assay (RIPA) buffer (Sigma Aldrich, St. Louis, MO, USA, #R0278) supplemented with cOmplete™ EDTA-free Protease Inhibitor Cocktail (Sigma Aldrich, #11873580001). Proteins were analyzed by 4%–12% Bis-Tris NuPAGE gels (Invitrogen, Carlsbad, CA, USA, #NP0322), transferred to PDVF membranes (GE Healthcare, Boston, MA, USA, #GE10600023), and probed with indicated antibodies. Rat anti-AID (1:500, Active Motif, Carlsbad, CA, USA, #39886); mouse anti-GCN5, clone A-11 (1:500, Santa Cruz Biotechnology, Dallas, TX, USA, #sc-365321); rabbit anti-PCAF, clone C14G9 (1:1000, Cell Signaling Technology, Leiden, The Netherlands, #3378); rabbit antihistone H3 (1:1000, Abcam, Cambridge, UK, #ab1791); rabbit antihistone H3 acetyl K9 (1:500, Abcam, Cambridge, UK, #ab32129).

### 2.5. Statistics

We performed statistical analyses with one-way ANOVA using GraphPad Prism 8.0.1.244 (San Diego, CA, USA). In the tests, *p* values less than 0.05 were defined as significant, i.e., * *p* < 0.05; ** *p* < 0.01; *** *p* < 0.001; and **** *p* < 0.0001.

## 3. Results

### 3.1. Generation of Mice Lacking GCN5 and PCAF in B Cells

The combined inactivation of *Gcn5* and *Pcaf* genes in mice results in embryonic lethality [32]. To overcome this challenge, we designed a complex genetic model when the floxed *Gcn5* gene is conditionally inactivated in B-cell lineages by the CRE enzyme under the *Cd19* promoter (*Cd19^Cre+^*) [34]. To sort out cells with activated CRE, we used a model with knocked-in yellow fluorescent protein gene (*YFP*) into the ROSA-26 locus. YFP is inactive until CRE removes the “STOP” signal (Rosa-26-YFP+) [35]. Thus, we obtained *Gcn5^f/f^Pcaf^−/−^Cd19^+/Cre^YFP^+^* mice and simpler controls. Further in the text, we skip *Cd19^+/Cre^* and *YFP+* for simplicity in most cases, and refer to mice on the basis of the status of *Gcn5* and *Pcaf* genes, i.e., as *Gcn5^f/f^Pcaf^−/−^*, *Gcn5^f/f^*, *Pcaf^−/−^*, and WT. When the CRE is active and describing sorted B cells, we indicate *Gcn5^−/−^*, a knockout status of the gene. The lack of GCN5 and PCAF, and H3K9 acetylation, were validated using Western blot (Figure S1).

### 3.2. Mice Lacking GCN5 and PCAF in B Cells Possess Small Spleens

**Figure 1:**
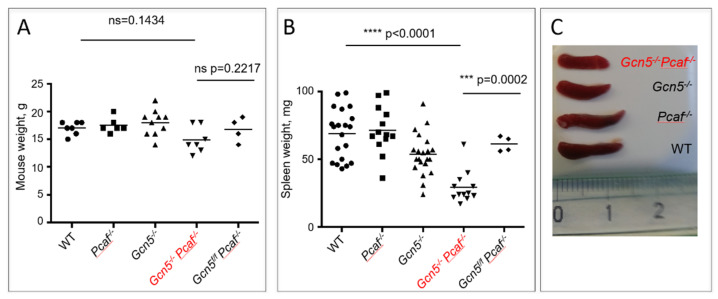
Generation of mice with germline inactivation of *Pcaf* and conditional inactivation of *Gcn5* in B-cell lineages. (**A**) Sizes of 8-week-old mice of indicated genotype were similar (*p* > 0.1433). (**B**) Size of spleens from mice of indicated genotypes. WT vs. *Pcaf^−/−^*, n.s. *p* = 0.9559; WT vs. *Gcn5^−/−^*, * *p* = 0.0192; WT vs. *Gcn5^−/−^Pcaf^−/−^*, **** *p* < 0.0001; *Pcaf^−/−^* vs. *Gcn5^−/−^*, * *p* = 0.0119; *Pcaf^−/−^* vs. *Gcn5^−/−^Pcaf^−/−^*, **** *p* < 0.0001; *Gcn5^−/−^* vs. *Gcn5^−/−^Pcaf^−/−^*, *** *p* = 0.0008. (**C**) Example of spleens of indicated genotypes. *Gcn5^−/−^* indicates *Cd19^Cre^*-dependent inactivation of *Gcn5* in B-cell lineages.

We obtained mice with the germline inactivation of *Pcaf* gene and conditional inactivation of *Gcn5* in B cells under the *Cd19* promoter (Figure 1). The germline inactivation of *Pcaf* gene alone had no detectable effect on mouse development, in line with a previous observation [32]. The conditional inactivation of the *Gcn5* gene in B cells had no visible effect on sizes of WT and *Pcaf*-deficient mice, which were 15 to 19 g on average (*p* > 0.1433) (Figure 1A). However, the inactivation of *Gcn5* resulted in smaller spleens in mice (*Gcn5^−/−^*, 54 mg) when compared to WT (69 mg) and *Pcaf^−/−^* (72 mg) mice. The combined inactivation of *Pcaf* and *Gcn5* in B cells resulted in even smaller spleens (*Gcn5^−/−^Pcaf^−/−^*, 29 mg, *p* < 0.0001). The spleens of mice without CRE activity with the *Gcn5* gene being floxed and functional (*Gcn5^f/f^Pcaf^−/−^*, 67 mg) were comparable in size to the ones of WT mice (Figure 1B,C).

### 3.3. Mice Lacking GCN5 and PCAF in B Cells Possess Delayed B Lymphocyte Development

**Figure 2:**
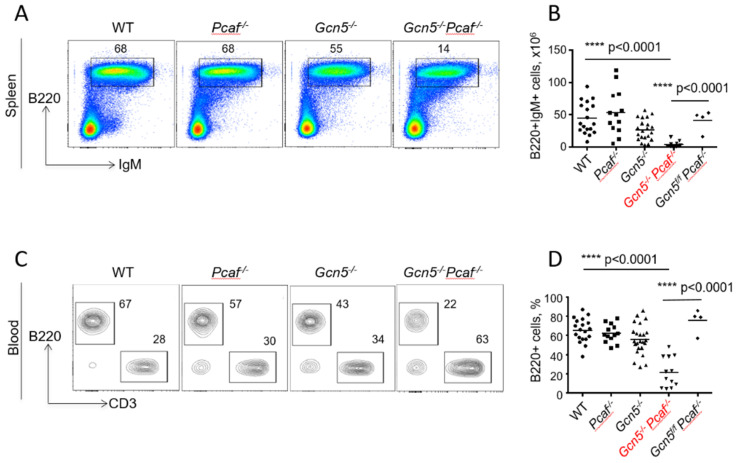
Reduced levels of mature B lymphocytes in spleens and blood of 8–12-week-old mice of indicated genotypes. (**A**) Proportions of B220+IgM+ mature B cells in spleen. WT vs. *Pcaf^−/−^*, n.s. *p* = 0.4777; WT vs. *Gcn5^−/−^*, ns, *p* = 0.2532; WT vs. *Gcn5^−/−^Pcaf^−/−^*, **** *p* < 0.0001; *Pcaf^−/−^* vs. *Gcn5^−/−^*, * *p* = 0.0119; *Pcaf^−/−^* vs. *Gcn5^−/−^Pcaf^−/−^*, **** *p* < 0.0001; *Gcn5^−/−^* vs. *Gcn5^−/−^Pcaf^−/−^*, *** *p* < 0.0001. (**B**) Summary of experiments as shown in (**A**), number of cells. WT vs. *Pcaf^−/−^*, n.s., *p* = 0.9212; WT vs. *Gcn5^−/−^*, * *p* = 0.0214; WT vs. *Gcn5^−/−^Pcaf^−/−^*, **** *p* < 0.0001; *Pcaf^−/−^* vs. *Gcn5^−/−^*, ** *p* = 0.0053; *Pcaf^−/−^* vs. *Gcn5^−/−^Pcaf^−/−^*, **** *p* < 0.0001; *Gcn5^−/−^* vs. *Gcn5^−/−^Pcaf^−/−^*, * *p* < 0.0269. (**C**) Proportions of B220+ B cells and CD3+ T cells in blood. (**D**) Summary of several experiments from (**C**) reflecting proportions of B cells in blood. WT vs. *Pcaf^−/−^*, n.s. *p* = 0.9196; WT vs. *Gcn5^−/−^*, n.s., *p* = 0.1204; WT vs. *Gcn5^−/−^Pcaf^−/−^*, **** *p* < 0.0001; *Pcaf^−/−^* vs. *Gcn5^−/−^*, n.s., *p* = 0.5398; *Pcaf^−/−^* vs. *Gcn5^−/−^Pcaf^−/−^*, **** *p* < 0.0001; *Gcn5^−/−^* vs. *Gcn5^−/−^Pcaf^−/−^*, *** *p* < 0.0001. *Gcn5^−/−^* and *Gcn5^−/−^Pcaf^−/−^* indicate *Cd19^Cre^*-dependent inactivation of *Gcn5* in B-cell lineages.

To detect mature *Gcn5^−/−^Pcaf^−/−^* B cells, we identified B220+IgM+ cells in the spleen using flow cytometry (Figure 2A,B). The inactivation of the *Pcaf* gene alone did not affect B-cell proportions in the spleen (58%) when compared to WT mice (52%, *p* = 0.4777) (Figure 2A). The inactivation of *Gcn5* alone resulted in an insignificant reduction in mature splenocytes when compared to WT mice (*Gcn5^−/−^*, 46%, *p* < 0.2532), although *Gcn5^−/−^Pcaf^−/−^* mice had significantly less B-cell frequency in the spleen (21%, *p* < 0.0001). Similarly, the number of *Gcn5^−/−^Pcaf^−/−^* B splenocytes was the lowest (3, 4 million), while the number of *Gcn5^−/−^* B cells (27 million) was also reduced when compared to *Pcaf^−/−^* (54 million, ** *p* = 0.0065) and WT (44 million, * *p* = 0.0214) controls (Figure 2B).

### 3.4. Inactivation of Gcn5 and Pcaf Results in a Reduced Proportion of B Cells in the Blood

To detect mature B cells in the blood, we used B220 markers (Figure 2C,D). The inactivation of *Pcaf* alone resulted in 62% of B cells after red blood cells had been lysed, which was comparable to WT mice with 65% of B cells in the blood (*p* = 0.92). The inactivation of the *Gcn5* gene alone resulted in a modest reduction of B-cell proportion to 56% (*p* = 0.12), while the combined inactivation of *Gcn5* and *Pcaf* led to even lower B-cell levels in the blood (22%, *p* < 0.0001). The levels of B cells in the blood of control mice without CRE recombinase expression when the *Gcn5* gene was functional (*Gcn5^f/f^Pcaf^−/−^*) were comparable to those of WT mice (78%). GCN5 and PCAF are thus both required and functionally redundant for B-cell development in mice.

**Figure 3:**
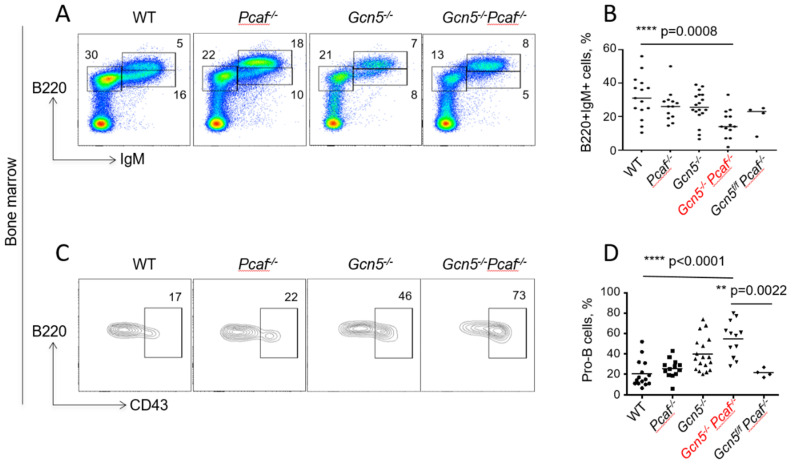
Delayed development of GCN5/PCAF-deficient B cells in bone marrow. Flow cytometry analyses of developing B lymphocytes in bone marrow of 8–12-week-old mice indicated genotypes. (**A**) Examples of B220+IgM− (pro-B and pre-B), B220+IgM+low (immature B), and B220+IgM+high (mature B) cell populations. (**B**) Summary of several experiments from (**A**) indicating B220+IgM+ cells. WT vs. *Pcaf^−/−^*, n.s. *p* = 0.6445; WT vs. *Gcn5^−/−^*, n.s., *p* = 0.4432; WT vs. *Gcn5^−/−^Pcaf^−/−^*, *** *p* = 0.0008; *Pcaf^−/−^* vs. *Gcn5^−/−^*, n.s., *p* = 0.9997; *Pcaf^−/−^* vs. *Gcn5^−/−^Pcaf^−/−^*, * *p* < 0.0138; *Gcn5^−/−^* vs. *Gcn5^−/−^Pcaf^−/−^*, * *p* < 0.0211 (**C**) CD43+ (pro-B cells) and CD43- (pre-B cells) gated from B220+IgM− populations in (**A**). (**D**) Summary of several experiments detecting B220+IgM−CD43+ (pro-B) cells. WT vs. *Pcaf^−/−^*, n.s. *p* = 0.7825; WT vs. *Gcn5^−/−^*, ** *p* = 0.0022; WT vs. *Gcn5^−/−^Pcaf^−/−^*, **** *p* < 0.0001; *Pcaf^−/−^* vs. *Gcn5^−/−^*, n.s., *p* = 0.0505; *Pcaf^−/−^* vs. *Gcn5^−/−^Pcaf^−/−^*, **** *p* < 0.0001; *Gcn5^−/−^* vs. *Gcn5^−/−^Pcaf^−/−^*, * *p* < 0.0322 *Gcn5^−/−^* and *Gcn5^−/−^Pcaf^−/−^* indicate *Cd19^Cre^*-dependent inactivation of *Gcn5* in B-cell lineages.

One reason for the low *Gcn5^−/−^Pcaf^−/−^* B-cell count in spleen and blood in mice could be cell death following the normal development of B cells in bone marrow and migration to the periphery. Another option could be the blocked or delayed maturation of B cells in bone marrow during the earlier developmental stages. To test the latter possibility, we analyzed B cells in the bone marrow of the mice (Figure 3).

### 3.5. Inactivation of Gcn5 and Pcaf Results in Accumulation of Pro-B Cells in Bone Marrow

To characterize B-cell maturation in bone marrow, we followed the expression of B220 (B220+IgM−) and IgM (B220+IgM+) on the lymphocyte surface. The inactivation of *Gcn5* or *Pcaf* resulted in an insignificant decline in the B220+IgM+ population (26%, 5–6 million) when compared to WT (32%, 8 million, *p* = 0.44) (Figure 3 A,B). The combined inactivation of *Gcn5* and *Pcaf* resulted in an additional reduction in mature B cells in bone marrow (3 million, 16%) (Figure 3A,B).

We further focused on B220+IgM− populations by determining the CD43+ (pro-B cells) and CD43- (pre-B cells). The proportion of early-stage pro-B cells increased from WT mice (20% on average) and *Pcaf^−/−^* mice (26%, *p* = 0.78) to *Gcn5^−/−^* (39%, ** *p* = 0.0022) to *Gcn5^−/−^Pcaf^−/−^* (55%, *p* < 0.0001) (Figure 3C,D). However, the number of pro-B cells in bone marrow, estimated using our method of cell extraction, was rather stable, with 0.8 million for WT (*n* = 13) and about 1.3 million for *Gcn5^−/−^* (*n* = 13), *Pcaf^−/−^* (*n* = 13), and *Gcn5^−/−^Pcaf^−/−^* (n = 5) cells (n.s., *p* > 0.3830). This suggested that the proportion of *Gcn5^−/−^Pcaf^−/−^* pro-B cells was increased because the total number of mature B cells was reduced (Supplemental Figure S3 and 3B).

We concluded that GCN5 and PCAF are required for the maturation of B cells from the pro-B to the pre-B cell stage, and later to mature B cells.

### 3.6. GCN5 Is Required for Robust Class Switch Recombination

**Figure 4:**
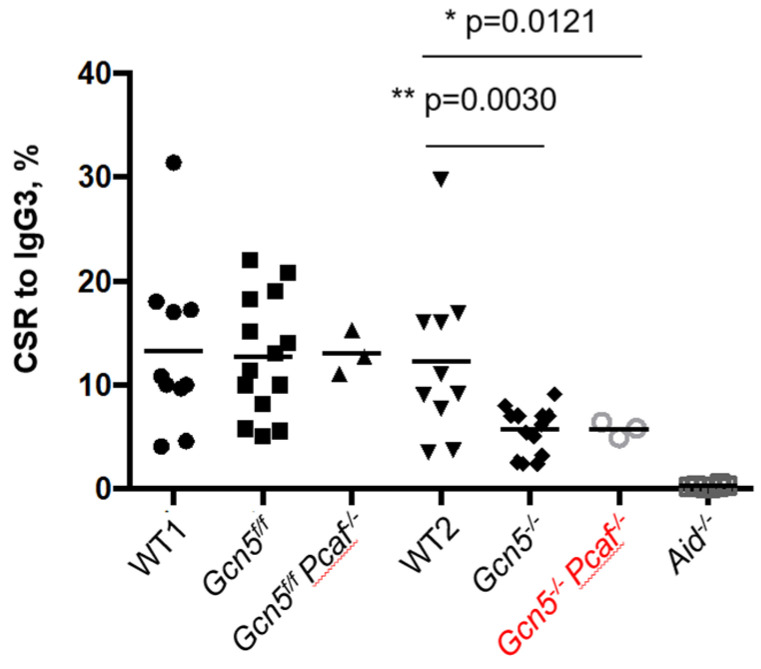
Class switch recombination of stimulated mature B cells from IgM to IgG3. *Gcn5^−/−^* and *Gcn5^−/−^Pcaf^−/−^* indicate *Cd19^Cre^*-dependent inactivation of *Gcn5* in B-cell lineages. Levels of CSR for WT, *Gcn5^f/f^*, *Gcn5^f/f^Pcaf^−/−^* were not significantly different (n.s.); levels for *Gcn5^−/−^* and *Gcn5^−/−^Pcaf^−/−^* were lower than those of the three former groups (** *p* = 0.0030 and * *p* = 0.0121, correspondently, when compared to WT2); *Gcn5^−/−^* vs. *Gcn5^−/−^Pcaf^−/−^* levels were similar (n.s., *p* = 0.9997), and *Aid^−/−^* only had background levels, WT2 vs. *Aid^−/−^* is (**** *p* > 0.0001).

The CSR relies on the ATM-dependent DDR [2,4,5,27]. Because H3K9 acetylation works downstream of H2AX phosphorylation, and GCN5/PCAF might work downstream of ATM/ATR/DNA-PKcs, we tested if the CSR depends on GCN5 and PCAF (Figure 4). We purified B splenocytes from 8- to 12-week-old mice and stimulated the CSR from IgM to IgG3 using established protocols [40,42]. We focused on matched pairs of *Gcn5^f/f^* (the functional equivalent of WT cells) and *Gcn5^−/−^*, as well as *Gcn5^f/f^Pcaf^−/−^* (the functional equivalent of *Pcaf^−/−^*) and *Gcn5^−/−^Pcaf^−/−^* cells. We used *Aid^−/−^* cells as a CSR-deficient control to detect an experimental background (Figure 4). The inactivation of *Pcaf* alone had no effect on CSR levels (WT vs. *Gcn5^f/f^Pcaf^−/−^*, *p* > 0.96). On the other hand, the inactivation of *Gcn5* resulted in a reduction in CSR from about 14% in WT and *Gcn5^f/f^* cells to 6% in *Gcn5^−/−^* cells, * *p* < 0.0008 (Figure 4). The combined deletion of *Pcaf* and *Gcn5* resulted in a similar reduction from 12% in *Gcn5^f/f^Pcaf^−/−^* cells to 6% in *Gcn5^−/−^Pcaf^−/−^* cells, ** *p* = 0.0080. We concluded that GCN5 is required for robust CSR to IgG3 because the additional inactivation of *Pcaf* did not affect CSR levels when compared to *Gcn5^−/−^* and *Gcn5^−/−^Pcaf^−/−^* B cells, *p* > 0.9999 (Figure 4).

## 4. Discussion

Both GCN5 and PCAF are involved in chromatin modification and DDR response, which made them relevant candidates to facilitate lymphocyte development [13,14]. One challenge was the lack of a relevant in vivo model because GCN5 and PCAF have certain redundant functions in acetylating H3K9, and because the germline inactivation of *Gcn5* results in early embryonic lethality in mice [32]. Here, we generated and analyzed a complex mouse model that allowed for studying of double-deficient *Gcn5^−/−^Pcaf^−^*^/−^ B cells development in vivo and ex vivo. We used a germline knockout of *Pcaf* [32], a conditional knockout of *Gcn5^f/f^*, a knockin of CRE recombinase expressed under the B-cell-specific *Cd19* promoter [34], and a knockin of YFP to track the activity of CRE recombinase [35].

For such a complex mouse model (*Gcn5^f/f^Pcaf^−/−^Cd19^+/cre^Rosa-26-YFP^+^*), multiple controls were used. In one line of the controls, mice lacking PCAF and having a floxed *Gcn5* gene but expressing no CRE recombinase were considered (*Gcn5^f/f^Pcaf^−/−^Rosa-26-YFP^+^*, Figure S2). The GCN5-deficient and GCN5/PCAF double-deficient B cells possessed developmental delay, with lower levels of mature B cells in thee spleen and blood, and the accumulation of progenitor B cells in bone marrow (Figure 1, Figure 2, Figure 3 and Figure 4). Control mice without CRE expression, on the other hand, demonstrated WT levels of B-cell development in all groups, i.e., WT levels of B220+IgM+ mature B cells in the spleen (Figure S2A,B) and blood (Figure S2C,D). In addition, these mice possessed high levels of B220+IgM+ cells (Figure S2E,F), and stable and low levels of pro-B cells (B220+IgM−CD43+) in bone marrow (Figure S2G,H).

Mice lacking PCAF and with conditional knockout of *Gcn5* in B cells were alive and resembled WT littermates (Figure 1A). One clear feature that the *Gcn5^f/f^Pcaf^−/−^Cd19^+/cre^Rosa-26-YFP^+^* mice had was a small spleen (Figure 1B,C), which was also the case in mice lacking only *Gcn5* in B cells. The small spleen could indicate a defect in B-cell development, and we indeed found low numbers of mature B cells in the spleen, blood, and bone marrow. One could propose that mature B cells lacking GCN5 or both GCN5 and PCAF possess low proliferation speed or tend to trigger apoptosis. Alternatively, GCN5 and PCAF might be required for the V(D)J recombination. This option could be tested by, for example, using vAbl pre-B cell lines, as we and others did before [4,15,18,23,27,28,29,30,31]. Another intriguing question is whether the physical presence or enzymatic activity of GCN5 and PCAF are required for the observed phenotypes, i.e., abrogated B-cell maturation and reduced levels of CSR. To investigate this question, one could use specific inhibitors of GCN5 and PCAF enzymes, or enzyme-dead mutations introduced to the *Gcn5* and *Pcaf* genes.

The inactivation of *Gcn5* in murine B cells also resulted in reduced lymphomagenesis in mice overexpressing MYC oncoprotein [44]. Our findings further highlight this observation, suggesting that GCN5 and potentially also PCAF enzymes are attractive targets for cancer therapy [44].

CSR levels were reduced in B cells lacking GCN5 (Figure 4). The challenge in this set of experiments was that mice of the *Gcn5^f/f^Pcaf^−/−^Cd19^+/cre^Rosa-26-YFP^+^* genotype were rather rare and possessed a very low number of suitable B splenocytes (Figure 1 and Figure 2). Although our data on IgG3 are sufficient, one could extend the study by generating knockout cell lines lacking GCN5 and PCAF, and suitable for CSR. One possible model system is CH12F3 cells capable of supporting CSR to IgA [45], which were used for this kind of experiment [24,37,42]. The CSR itself is a complex multistage process. Generating relevant cell lines also provides tools to determine specific stages of CSR affected in GCN5-deficient mice, i.e., germline transcription, AID recruitment, generation of DSBs, or DNA repair.

## 5. Conclusions

Acetyltransferases GCN5 and PCAF possess redundant functions in B-cell maturation. GCN5 is required for robust class switch recombination ex vivo.

## Data Availability

The data presented in this study are available in the main text, figures, tables and Supplementary Materials.

## Supplementary Materials

The following supporting information can be downloaded at: https://www.mdpi.com/article/10.3390/biom12010061/s1, Figure S1: Detection of GCN5, PCAF and histones; Figure S2: Detection of developing B cells in mice of indicated genotypes using flow cytometry; Figure S3: Count of WT, Pcaf-deficient, Gcn5-deficient, and Gcn5/Pcaf double-deficient pro-B cells in bone marrow.
