# Supplementary material for: Acetyltransferases GCN5 and PCAF Are Required for B Lymphocyte Maturation in Mice
Source: Biomolecules. 2021 Dec 31;12(1):61. doi: 10.3390/biom12010061 (PMC8773862; doi:10.3390/biom12010061)

Aceltransferases GCN5 and PCAF are required for B Lymphocyte maturation in mice

Supplementary Figure S1. Detection of GCN5, PCAF and histones using western blot

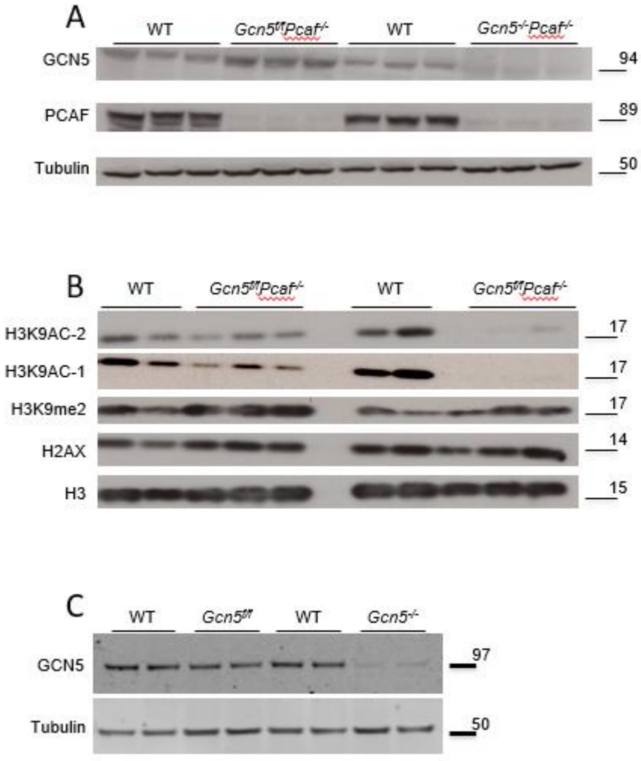

Supplementary Figure S1. Detection of GCN5, PCAF and histones.
(A) Detection of GCN5 and PCAF in primary murine B cells of indicated genotype. GCN5 signal is absent in the *Gcn5*$^{-/-}$ cells. PCAF signal is absent in the *Pcaf*$^{-/-}$ cells. Tubulin was used as a loading control.
(B) Western blot detecting Histones H3, H2AX, H3K9me2, H3K9ac (two different antibodies). H3K9Ac signal is missing in the cells lacking both GCN5 and PCAF.
(C) Detection of GCN5 and PCAF in primary murine B cells of indicated genotype. GCN5 signal is absent in the *Gcn5*$^{-/-}$ cells.

Supplementary Figure S2. Detection of developing B cells in mice of indicated genotypes using flow cytometry

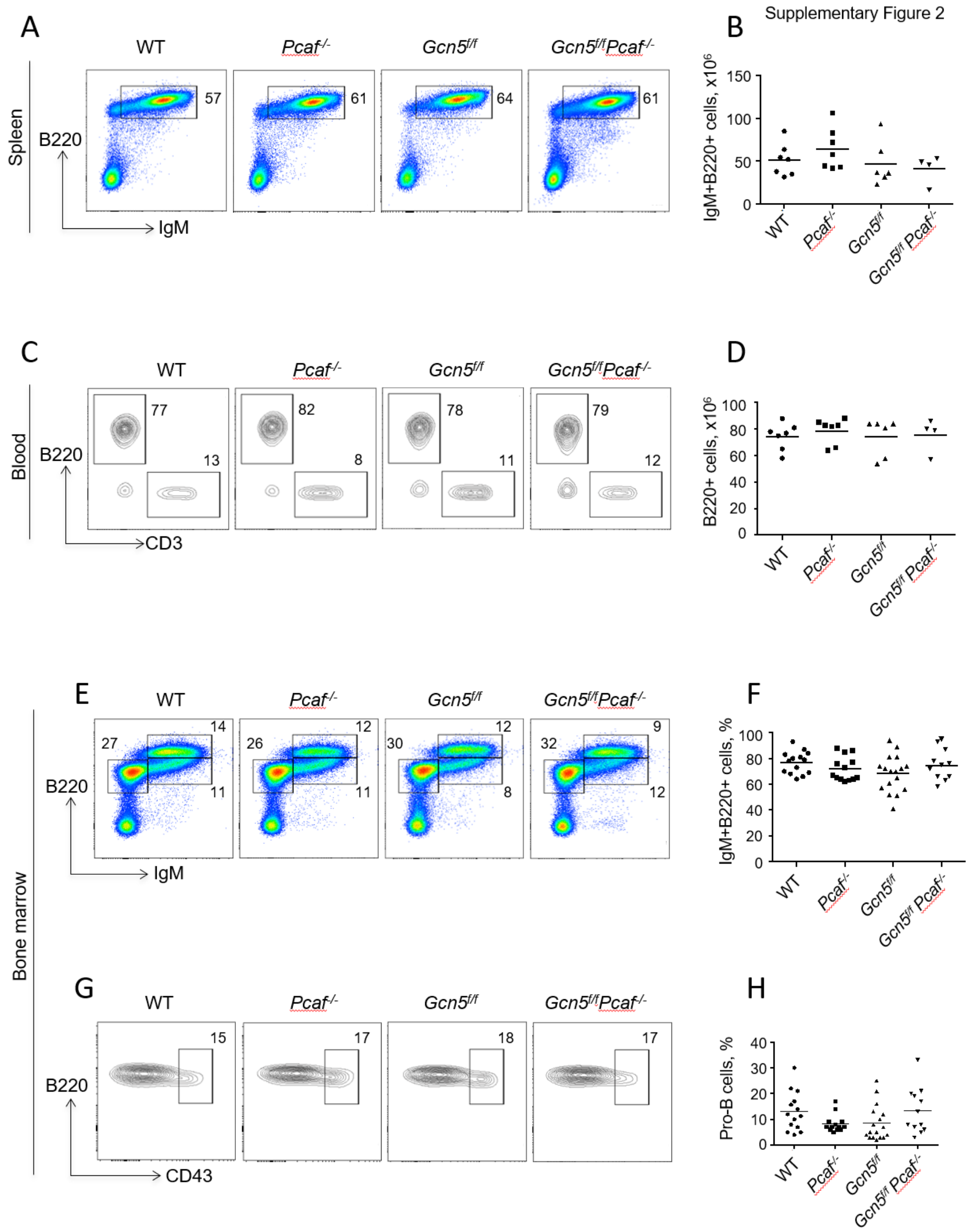

Supplementary Figure S2. Detection of developing B cells in mice of indicated genotypes using flow cytometry

(A) Example of flow cytometry detecting B220+IgM+ mature B cells in spleen.
(B) Summary of several experiments shown in (A).
(C) Example of flow cytometry detecting B220+ B cells and CD3+ T cells in blood.
(D) Summary of several experiments shown in (C).
(E) Example of flow cytometry detecting B220+IgM- and B200+IgM+ developing B cells in bone marrow.
(F) Summary of several experiments shown in (E).
(G) Example of flow cytometry detecting B220+IgM-CD43+ pro-B cells and B220+IgM-CD43- pre-B cells in bone marrow.
(H) Summary of several experiments shown in (G).

Supplementary Figure S3. Count of WT, *Pcaf*-deficient, *Gcn5*-deficient, and *Gcn5*/*Pcaf* double-deficient pro-B cells in bone marrow (million cells); n.s. p>0.3830

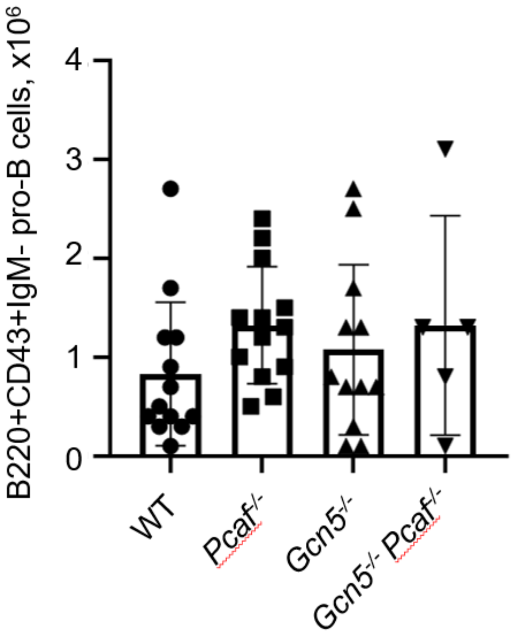

Supplement: Supplementary file 1 [file biomolecules-12-00061-s001.zip › biomolecules-1528668-supplementary.pdf]
